# Sociocultural determinants of attitudes toward oocyte donation among infertility patients in Western Iran

**DOI:** 10.1038/s41598-026-45147-3

**Published:** 2026-03-29

**Authors:** Masoumeh Esmaeilivand, Shokoh Jahanbakhsh, Shahab Rezaeian, Alireza Khatony

**Affiliations:** 1 Clinical Research Development Center, Motazedi Hospital, Reproductive Health, Kermanshah University of Medical Sciences, Kermanshah, Iran; 2 Department of Obstetrics and Gynecology, School of Medicine, Kermanshah University of Medical Sciences, Kermanshah, Iran; 3 Student Research Committee, Kermanshah University of Medical Sciences, Kermanshah, Iran; 4 School of Health, Kermanshah University of Medical Sciences, Kermanshah, Iran; 5 Social Development and Health Promotion Research Center, Kermanshah University of Medical Sciences, Kermanshah, Iran

**Keywords:** Oocyte donation, Attitudes, Anonymity, Disclosure, Reproductive ethics, Iran, Diseases, Genetics, Health care, Medical research, Risk factors

## Abstract

Attitudes toward oocyte donation in Iran are deeply shaped by sociocultural forces, including concepts of lineage, stigma, privacy, and gendered expectations. Evidence from western Iran remains limited. This study examined attitudes toward oocyte donation and their sociocultural determinants among infertility patients. This cross-sectional survey was conducted among 171 infertile men and women attending a public infertility center in western Iran. Data were collected using a validated 52-item multidimensional attitude questionnaire distributed as a self-administered paper survey in a clinical setting. Multivariable linear regression was used to assess associations between sociodemographic characteristics and attitude domains. Participants demonstrated generally positive attitudes toward oocyte donation (mean = 177.21 ± 26.15). However, clear sociocultural patterns emerged, with strong endorsement of donor–recipient anonymity, secrecy from others, and non-disclosure to the future child, reflecting concerns related to lineage and social judgment. Higher male educational level was significantly associated with stronger support for anonymity, while female education showed a modest association with disclosure-related attitudes. Attitudes toward oocyte donation in western Iran are shaped primarily by sociocultural expectations surrounding privacy, secrecy, and lineage rather than clinical characteristics. These findings highlight the importance of culturally sensitive counseling and education strategies in donor-assisted reproduction programs.

## Introduction

Infertility is a significant global health concern, affecting approximately one in six adults worldwide, according to recent estimates from the World Health Organization^1^. In Iran, the prevalence of primary and secondary infertility has been estimated at 18.3% and 2.5%, respectively, highlighting both the clinical burden and the growing demand for effective fertility care^2^. Both global and national estimates are presented as prevalence measures, using the most commonly reported formats in the respective sources.

Advances in assisted reproductive technologies (ART) have expanded treatment options for individuals and couples with infertility, including those requiring third-party gametes. Oocyte donation has become an established therapeutic option worldwide and accounts for an increasing proportion of ART cycles^3^. However, acceptance of oocyte donation varies widely and is shaped by social norms, gender expectations, religious principles, and legal frameworks. Recent work on the Ethical, Legal, and Social Implications (ELSI) of gamete donation emphasizes persistent tensions around anonymity, disclosure, identity, and power relations between donors, recipients, and offspring^4^.

The Middle East provides a particularly important context for studying these issues. In many Muslim-majority countries, donor-assisted reproduction intersects with religiously grounded concepts of lineage (nasab), marital fidelity, and social legitimacy. Studies from Iran and other Middle Eastern countries have documented concerns about stigma, secrecy, and the potential destabilization of marital and kinship relations following donor conception^5^. In Iranian society, confidentiality in donor conception is not merely an individual preference but a socially enforced norm. Concerns about family honor, lineage preservation (nasab), and social labeling strongly shape attitudes toward secrecy and non-disclosure in infertility treatment. Unlike many Muslim-majority countries, Iran permits oocyte donation under Shi’a jurisprudence through specific legal and religious frameworks, creating a unique coexistence of formal permissibility and informal social resistance.

Iran is unique in that oocyte donation is legally and religiously permitted under specific conditions, and ART centers are widely operational. Yet, cultural and ethical barriers continue to influence decision-making. Earlier Iranian studies have reported heterogeneous attitudes toward oocyte donation, ranging from viewing it as a “last resort” to relatively high acceptance among some groups of infertile women. Qualitative work has highlighted women’s fears regarding marital stability, the child’s future identity, and social judgment, as well as the strategic use of secrecy to “normalize” donor conception within families^6^. Reliable national prevalence data on oocyte donation in Iran are limited, partly due to confidentiality practices and the absence of centralized reporting systems. More recent Iranian studies have also shown that intention to use oocyte donation is influenced by attitudes, subjective norms, and perceived behavioral control, suggesting that sociocultural and gendered expectations are central to reproductive decision-making^7^.

Despite this growing body of evidence, important gaps remain. First, most empirical studies in Iran have focused on central or northeastern provinces, with limited data from western regions such as Kermanshah, where cultural, religious, and ethnic diversity may shape reproductive attitudes differently. Western Iran is characterized by ethnic, cultural, and religious diversity, which may intensify concerns related to social visibility, disclosure, and family reputation in the context of donor-assisted reproduction. Second, few studies have quantitatively examined how sociodemographic characteristics particularly male versus female education relate to specific domains of attitude, such as anonymity, disclosure to the child, and perceptions of the parent–child relationship. Third, much of the global ELSI literature on gamete donation is derived from Western contexts, with limited integration of perspectives from Muslim-majority societies^4^.

To address these gaps, the present study aimed to evaluate attitudes toward oocyte donation among infertility patients in western Iran using a validated, multidimensional questionnaire developed in the Iranian context. Specifically, we sought (1) to describe overall and domain-specific attitudes particularly regarding anonymity, secrecy, and the parent–child relationship and (2) to identify sociodemographic predictors of these attitudes, with a focus on gender and educational level. By generating region-specific evidence, this study contributes to a more nuanced understanding of how sociocultural determinants shape acceptance of oocyte donation and can inform culturally responsive counseling and policy-making in donor-assisted reproduction programs.

## Materials and methods

This cross-sectional study with multivariable regression analysis was conducted between October 2023 and January 2024 at Motazedi Hospital, a public infertility center in Kermanshah, western Iran. The center provides comprehensive assisted reproductive services and attracts a diverse patient population from across the region, offering an appropriate setting for examining individual attitudes toward oocyte donation. The study was designed and reported in accordance with STROBE guidelines^8^.

### Participants and recruitment

Participants were adults (≥ 18 years) with a history of at least one year of infertility who attended the center during the study period. Individuals were eligible if they were able to read and complete the questionnaire independently. Those who returned incomplete questionnaires were excluded. Eligibility criteria were defined for participation in an attitude assessment study and do not reflect clinical eligibility for oocyte donation treatment.

Recruitment was conducted through a consecutive sampling approach, whereby potentially eligible patients were approached in the waiting area and invited to participate. To maintain analytical independence and avoid dyadic clustering, only one partner from each couple was included; responses therefore represent individual rather than joint attitudes. Accordingly, the term “patients” is used throughout the manuscript to reflect individual-level responses rather than joint couple perspectives. All participants provided written informed consent prior to data collection.

Sample size was estimated using parameters from Jafari et al.^9^, incorporating mean and standard deviation values of attitude scores, a 5% type I error, and a 15% adjustment for variance. The required sample of 171 participants was successfully obtained.

### Measures

Attitudes toward oocyte donation were assessed using the validated 52-item Attitude Toward Oocyte Donation Questionnaire, originally developed and psychometrically tested within the Iranian context^10,11^. The questionnaire comprises 12 domains that address key aspects of oocyte donation, including decision-making, donor and recipient characteristics, anonymity, disclosure, legal issues, and perceptions of the parent–child relationship and belonging.

Items are rated on a 5-point Likert scale (1 = strongly disagree to 5 = strongly agree). Domain scores are calculated as the sum of item responses within each domain, with higher scores indicating more favorable attitudes. Internal consistency of the overall scale and individual domains has been previously reported during the original validation of the instrument^10^.

Demographic and clinical data including age, sex, educational level, infertility type, infertility factor, infertility duration, and history of ART use were collected through self-report.

### Data collection procedures

Data were collected using a self-administered paper questionnaire distributed in a private area of the clinic to minimize social desirability bias. A trained researcher provided uniform explanations regarding study objectives, ensured privacy, and remained available for questions without influencing responses. Completing the questionnaire required approximately 10–15 min. No identifying information was recorded.

### Statistical analysis

Analyses were conducted using STATA version 14. Descriptive statistics (means, standard deviations, frequencies, and percentages) were used to summarize demographic characteristics and attitude scores. Gender differences and comparisons across subgroups (e.g., ART history) were examined using independent-samples t-tests.

To identify predictors of attitudes, multivariable linear regression models were fitted for both the total attitude score and each of the 12 subscales. Female age, male age, female and male educational level, infertility type, infertility factor, and prior ART use were entered as covariates. Results are presented as unstandardized coefficients (b), 95% confidence intervals, and p-values. Statistical significance was defined as *p* < 0.05.

### Ethical considerations

Ethical approval for the study was obtained from the Ethics Committee of Kermanshah University of Medical Sciences (IR.KUMS.REC.1402.275). Participation was voluntary, and written informed consent was obtained from all participants. All methods were carried out in accordance with relevant guidelines and regulations. All procedures adhered to the principles of confidentiality and anonymity.

## Results

A total of 171 infertility patients participated in the study. Most respondents were women (68.42%), and the majority had educational attainment above a diploma level (84.11%). Primary infertility was more prevalent than secondary infertility (60.95% vs. 39.05%). The most frequently reported infertility etiology was female factor (40.91%), followed by combined factor (33.52%) and male factor (25.57%). Approximately one-third of participants (35.67%) reported previous experience with at least one ART procedure, and 36.26% indicated that they had considered or used oocyte donation at some point. These characteristics reflect a heterogeneous but clinically representative infertility population in western Iran. (Table 1)

**Table 1:** Demographic characteristics of participants.

Variable	Subgroup	Number	%
Gender	Males	54	31.57
	Females	117	68.42
Education	=< Diploma	27	15.88
	> Diploma	143	84.11
Type of infertility	Primary	103	60.95
	Secondary	66	39.05
Infertility factor	Male	45	25.57
	Female	72	40.91
	Male& Female	59	33.52
ART history	none	110	64.32
	At least one of them	61	35.67
History of Using oocyte donation method	No	109	63.74
	Yes	62	36.26

### Overall attitudes toward oocyte donation

Participants demonstrated generally favorable attitudes toward oocyte donation, with a mean total score of 177.21 ± 26.15, notably higher than the theoretical midpoint of 156. This indicates an overall positive orientation toward donor-oocyte treatment among infertility patients in this region. No significant difference was observed between women and men in the total score (178.07 ± 24.46 vs. 175.35 ± 29.62; *p* = 0.52), suggesting broadly similar levels of acceptance across genders. (Table 2)

### Domain-specific patterns of attitudes

Examination of the 12 attitude domains revealed a nuanced attitudinal profile. Scores related to decision-making and acceptance (21.17 ± 5.13) were moderately positive, reflecting a willingness to consider oocyte donation while still navigating uncertainties. Domain scores are not directly comparable, as each domain includes a different number of items and score ranges.

Attitudes toward donor and recipient characteristics were similarly positive, with participants expressing support for thoughtful matching and recognizing the importance of psychosocial considerations in donor selection.

Across domains addressing anonymity and disclosure, clear patterns emerged. Participants strongly favored both donor–recipient anonymity and child-to-donor anonymity, with domain means of 11.75 ± 2.73 and 8 ± 1.35, respectively. Likewise, attitudes reflected a preference for secrecy toward extended family and acquaintances, with limited support for disclosing the use of donor oocytes to others or to the future child. This cluster of responses points to deeply held cultural concerns surrounding privacy, lineage, and social judgment.

In contrast, participants reported highly favorable attitudes in the domains related to parent–child relationship (15.84 ± 3.55) and belonging (5.98 ± 1.56), indicating strong confidence in the quality of parental bonds regardless of genetic relatedness. These responses suggest that while genetic link is culturally meaningful, it does not diminish expectations of emotional closeness or parental identity. (Table 2)

**Table 2:** Attitudes of infertility patients across domains.

Domain	Total			Non using		Using		*p*- value	Male		Female		*p*- value
	Mean ± SD	Min	Max	Mean	SD	Mean	SD		Mean	SD	Mean	SD	
Attitude toward decision-making on oocyte donation	21.17 ± 5.13	7	35	20.90	5.11	21.65	5.16	0.36	21.68	4.99	20.93	5.19	0.37
Attitude toward roles of donors	14.52 ± 5.31	5	25	14.24	5.42	15.01	5.12	0.36	14.05	5.67	14.74	5.15	0.43
Attitude toward an oocyte recipient	30.99 ± 6.16	8	40	31.35	6.66	30.34	5.14	0.30	30.20	7.01	31.36	5.72	0.25
Attitude toward an oocyte donors	30.84 ± 6.75	8	40	31.22	6.96	30.18	6.36	0.33	30.01	7.33	31.23	6.46	0.27
Attitude toward anonymity of donors and recipient	11.75 ± 2.73	3	15	11.91	2.62	11.45	2.92	0.29	11.31	2.82	11.95	2.68	0.15
Attitude toward anonymity of children to donors	8.87 ± 4.18	4	20	9.06	4.12	8.52	4.30	0.42	9.25	4.40	8.68	4.08	0.41
Attitude toward disclosure or secrecy of oocyte donation	11.58 ± 3.23	2	15	11.51	3.21	11.72	3.28	0.68	11.55	3.13	11.60	3.29	0.92
Attitude toward legal issues	11.44 ± 3.13	4	20	11.62	3.19	11.11	3.03	0.31	11.35	3.30	11.48	3.07	0.80
Attitude toward desire of various oocyte donation method	6.18 ± 2.04	2	10	6.10	2.06	6.32	2.01	0.48	6.24	2.20	6.15	1.97	0.80
Attitude toward parent –child relationship	15.84 ± 3.55	3	20	15.76	3.54	16	3.60	0.67	15.59	3.51	15.96	3.58	0.52
Attitude toward belonging	5.98 ± 1.56	2	10	5.98	1.53	6	1.63	0.94	6.16	1.64	5.90	1.52	0.31
Attitude toward child	8 ± 1.35	2	10	8.00	1.22	7.98	1.57	0.90	7.90	1.24	8.03	1.41	0.54
Total scale	177.21 ± 26.15	66	223	177.70	27.24	176.32	24.27	0.74	175.35	29.62	178.07	24.46	0.52

### Predictors of attitudes toward oocyte donation

Multivariable linear regression analysis showed that educational level was associated with specific attitude domains rather than overall attitudes. Higher male educational level was associated with stronger endorsement of donor–recipient anonymity (b = 0.49, 95% CI: −0.00 to 1.72; *p* = 0.05). Female educational level showed a modest association with disclosure-related attitudes (*p* = 0.03). No significant relationships were observed for age, infertility type, infertility factor, or ART history with the total attitude score. (Table 3)

**Table 3:** Factors associated with attitudes of infertility patients toward oocyte donation.

	Age female				Age male				Education female				Education male				Reason				Type			
	b	95% CI		P-value	b	95% CI		P-value	b	95% CI		P-value	b	95% CI		P-value	b	95% CI		P-value	b	95% CI		P-value
		LB	UB			LB	UB			LB	UB			LB	UB			LB	UB			LB	UB	
decision making	-0.09	-0.2	0.03	0.12	-0.03	-0.14	0.07	0.52	0.33	-0.50	1.17	0.43	-0.52	-1.3	0.30	0.21	-0.53	-2.09	1.03	0.50	-1.48	-3.07	0.09	0.06
Roles of donors	-0.04	-0.1	0.08	0.49	-0.00	-0.11	0.10	0.96	0.85	-0.00	1.72	0.05	0.00	-0.8	0.86	0.98	-0.32	-1.94	1.30	0.69	-0.83	-2.49	0.82	0.32
Oocyte recipient site	0.09	-0.0	0.23	0.19	0.06	-0.06	0.19	0.30	-0.03	-1.05	0.97	0.93	0.10	-0.8	1.11	0.82	-0.39	-2.28	1.50	0.68	0.86	-1.06	2.78	0.37
Oocyte donors site	0.42	-0.1	0.20	0.59	0.07	-0.06	0.21	0.29	0.13	-0.97	1.24	0.80	0.23	-0.8	1.32	0.67	-0.59	-2.67	1.47	0.57	0.44	-1.66	1.47	0.67
Anonymity of donors and recipients	0.01	-0.0	0.06	0.60	0.00	-0.04	0.06	0.73	0.37	-0.06	0.82	0.09	0.49	0.05	0.92	*0.02*	-0.74	-1.57	0.08	0.07	0.41	-0.43	1.27	0.33
Anonymity of children to donors	-0.08	-0.1	0.01	0.09	-0.0	-0.1	0.00	0.07	-0.28	-0.97	0.39	0.41	-0.22	-0.9	0.45	0.50	-0.93	-2.21	0.34	0.15	-1.26	-2.56	0.02	0.05
disclosure	-0.00	-0.0	0.07	0.88	-0.02	-0.08	0.04	0.51	0.57	0.05	1.10	0.03	0.39	-0.1	0.91	0.13	0.04	-0.95	1.03	0.93	-0.37	-1.38	0.63	0.46
Legal issues	-0.00	-0.0	0.07	0.98	0.00	-0.05	0.07	0.81	-0.33	-0.85	0.17	0.19	-0.43	-0.9	0.06	0.08	-0.48	-1.44	0.47	0.32	-0.25	-1.23	0.72	0.60
Desire	-0.00	-0.0	0.04	0.95	-0.00	-0.04	0.04	0.94	-0.17	-0.51	0.15	0.30	-0.11	-0.4	0.21	0.47	0.01	-0.61	0.63	0.97	0.13	-0.50	0.77	0.67
Parent – child relationship	0.06	-0.0	0.15	0.10	0.03	-0.04	0.10	0.38	0.33	-0.24	0.92	0.25	-0.03	-0.6	0.54	0.91	-0.27	-1.36	0.81	0.62	0.05	-1.05	1.16	0.92
belonging	0.00	-0.0	0.03	0.97	0.00	-0.02	0.03	0.76	0.03	-0.21	0.29	0.77	-0.05	-0.3	0.19	0.66	-0.22	-0.70	0.25	0.35	-0.30	-0.79	0.18	0.22
child	0.02	-0.0	0.05	0.10	0.00	-0.01	0.03	0.56	1.84	-0.22	0.22	1.00	-0.03	-0.2	0.18	0.76	-0.05	-0.47	0.35	0.78	-0.14	-0.57	0.27	0.48
Attitude	0.0	-0.5	0.6	0.94	0.06	-0.4	0.60	0.81	1.8	− 2.4	6.11	0.40	-0.1	-4.4	4.05	0.92	-4.51	-12.5	3.49	0.26	-2.76	-10.9	5.41	0.50

## Discussion

The present study provides new evidence on how sociocultural norms shape attitudes toward oocyte donation among infertility patients in western Iran. Although participants expressed generally positive attitudes toward using donor oocytes, their responses reflected a complex negotiation between medical possibilities and deeply rooted cultural expectations. This duality, the acceptance of treatment on one hand and the insistence on secrecy and anonymity on the other, aligns strongly with prior research across Middle Eastern societies. Studies have consistently shown that donor conception intersects with concerns about lineage (nasab), marital stability, and the preservation of family honor, making nondisclosure a common coping strategy among couples^5,10,12^.

One of the most prominent findings in our study was the strong endorsement of anonymity both between donors and recipients and between children and donors. This preference mirrors regional research indicating that many Iranian and Middle Eastern couples perceive disclosure as a threat to family cohesion or future child well-being^6,11,13^. Recent analyses of Ethical, Legal, and Social Implications (ELSI) frameworks emphasize that secrecy often reflects broader societal pressures rather than individual psychological motives^4,14^. Inhorn and colleagues argue that in Muslim-majority contexts, lineage and patrilineal continuity remain powerful cultural forces, shaping how donor conception is morally and socially interpreted^5^. Our results reinforce this interpretation, showing that even among individuals seeking treatment, concerns about social judgment and identity remain highly influential.

Despite these concerns, participants strongly affirmed emotional parenthood and the possibility of establishing a secure parent–child bond regardless of genetic ties. This finding is consistent with longitudinal studies from non-anonymous systems showing that donor-conceived families often develop healthy relational dynamics, especially when supported by counseling and appropriate follow-up services^14–16^. The coexistence of emotional acceptance and secrecy in our sample underscores the need for culturally adapted psychosocial support counseling that respects local values while addressing long-term implications for children and families.

Educational level emerged as a relevant sociocultural factor influencing specific attitude domains rather than overall attitudes toward oocyte donation. Higher male educational level was associated with stronger endorsement of donor–recipient anonymity, suggesting heightened sensitivity to issues of privacy, lineage, and social visibility. Previous Iranian behavioral models have similarly reported that subjective norms and perceived social pressure strongly influence reproductive decision-making among men^7,17^. In contrast, female educational level showed a modest association with disclosure-related attitudes, which may reflect women’s greater engagement with emotional and relational aspects of infertility and family communication. These distinctions highlight the importance of couple-centered counseling that acknowledges men’s and women’s different social positions, responsibilities, and fears.

Notably, no associations were observed between attitudes and infertility type, etiology, or prior ART experience. This supports research suggesting that in Middle Eastern contexts, sociocultural norms not clinical variables are the primary drivers of reproductive attitudes^6,10,12^. Even patients with extensive ART experience remain influenced by concerns about secrecy, stigma, and social legitimacy.

Overall, our findings emphasize that oocyte-donation acceptance in western Iran cannot be viewed merely as a medical or individual choice. Rather, it reflects a layered interplay of cultural expectations, religious narratives, gender roles, and social pressures. Policy frameworks and clinical counseling models must therefore move beyond biomedical considerations to address confidentiality, disclosure dilemmas, and culturally mediated fears about lineage. Integrating insights from both ELSI literature and local qualitative evidence will enable ART programs to provide more ethical, informed, and psychologically supportive care.

These findings carry important implications for reproductive-health practice in Iran. Clinicians and counselors should recognize that patients’ concerns about secrecy, anonymity, and lineage preservation are not simply personal preferences but reflect broader community expectations and long-standing cultural narratives. Incorporating structured counseling sessions that openly address disclosure dilemmas, gendered expectations, and long-term family dynamics may help couples navigate these concerns more confidently. Moreover, policymakers should consider developing regionally sensitive guidelines that acknowledge cultural diversity across different provinces, rather than relying on uniform national recommendations.

### Strengths and limitations

This study has several strengths, including the use of a validated, culturally grounded multidimensional questionnaire and the inclusion of both male and female infertility patients from a public referral center in western Iran. By examining multiple attitude domains simultaneously, the study provides a nuanced view of sociocultural patterns related to oocyte donation.

Several limitations should be considered when interpreting the findings. First, data were collected using self-report questionnaires, which may be subject to social desirability bias, particularly in culturally sensitive domains such as secrecy, guilt, and disclosure. Religious and social norms may have influenced participants’ willingness to openly express certain attitudes. Second, the sample included a higher proportion of women than men, which may limit the generalizability of gender-specific findings. Finally, as a single-center cross-sectional study conducted in western Iran, the results may not be fully generalizable to other regions of the country or to different sociocultural contexts.

## Conclusion

This study shows that infertility patients in western Iran generally hold favorable views toward oocyte donation, yet their acceptance remains strongly shaped by sociocultural norms surrounding lineage, privacy, and nondisclosure. While participants expressed confidence in the emotional legitimacy of parenthood regardless of genetic ties, they simultaneously emphasized the need for anonymity and secrecy particularly toward extended family and the future child. These patterns indicate that cultural considerations, rather than clinical factors, play the dominant role in shaping attitudes. The gender-specific influence of male educational level further highlights the importance of incorporating culturally and socially informed perspectives into counseling and decision-making processes. Programs and policies related to donor-assisted reproduction in Iran should therefore prioritize culturally sensitive communication strategies, address disclosure dilemmas, and support couples in navigating the sociocultural implications of oocyte donation. These findings suggest the potential value of culturally adapted infertility education programs, particularly those addressing male perspectives on privacy, anonymity, and disclosure within the Iranian sociocultural context.

## Data Availability

The data that support the findings of this study are available on request from the corresponding author. The data are not publicly available due to privacy or ethical restrictions.
